# Automating fractional flow reserve (FFR) calculation from CT scans: A rapid workflow using unsupervised learning and computational fluid dynamics

**DOI:** 10.1002/cnm.3559

**Published:** 2021-12-27

**Authors:** Neeraj Kavan Chakshu, Jason M. Carson, Igor Sazonov, Perumal Nithiarasu

**Affiliations:** ^1^ Biomedical Engineering Group, Zienkiewicz Centre for Computational Engineering Faculty of Science and Engineering, Swansea University Swansea UK

**Keywords:** automation, computational fluid dynamics, computer vision, coronary system, fractional flow reserve, passive digital twin, vessel segmentation

## Abstract

Fractional flow reserve (FFR) provides the functional relevance of coronary atheroma. The FFR‐guided strategy has been shown to reduce unnecessary stenting, improve overall health outcome, and to be cost‐saving. The non‐invasive, coronary computerised tomography (CT) angiography‐derived FFR (cFFR) is an emerging method in reducing invasive catheter based measurements. This computational fluid dynamics‐based method is laborious as it requires expertise in multidisciplinary analysis of combining image analysis and computational mechanics. In this work, we present a rapid method, powered by unsupervised learning, to automatically calculate cFFR from CT scans without manual intervention.

## Graphical abstract

Fractional flow reserve (FFR) provides the functional relevance of coronary atheroma. The FFR guided strategy has been shown to reduce unnecessary stenting, improve overall health outcome, and to be cost‐saving. Coronary computerised tomography (CT) angiography‐derived FFR (cFFR) is an emerging non‐invasive method in reducing invasive catheter based FFR measurements. In this work, we present a rapid method, powered by unsupervised learning, to automatically calculate (cFFR) from CT scans with no manual intervention.

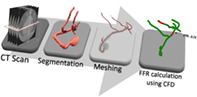

## 1 INTRODUCTION

In 2019, 218,032 people in the United Kingdom were affected by coronary heart disease (CHD) and a 63,237 people died as a result of it.^1^ These figures, however, reflect public health before the onset of COVID‐19 pandemic. The living conditions as a result of this pandemic has in fact increased the risk of mortality. In Great Britain, 9 in 10 coronavirus deaths had a pre‐existing condition and CHD was one of the most common ones.^2^ Further, with over 7.6 million living with cardiovascular diseases in the United Kingdom and ever growing waiting lists, stress on healthcare is expected to increase astronomically. A rapid and automatic screening for functional relevance of coronary stenoses is one of the potential solutions for easing this situation. With such screening, patients with advanced deterioration of coronary haemodynamic state can be prioritised, thereby reducing mortalities.

Until recently, coronary computerised tomography angiography (CCTA) was a widely adopted screening tool for coronary artery disease (CAD). However, detection of lesions and their severity, on its own, is insufficient to determine their functional relevance in oxygen supply to the cardiac tissue. Currently, invasive coronary catheter angiography‐based measurements of fractional flow reserve (FFR) have become the gold standard for the functional assessment of coronary artery obstructive lesions. The care planning, based on FFR, has shown to reduce unnecessary stenting and improve overall health of the patient.^3^

Although measuring FFR is beneficial, its invasive nature comes with challenges. In terms of procedure, time involved and expertise required makes it extremely laborious and slow. A risk of failure, sometimes fatal in nature, is also present in this procedure. About 0.05% patients lose their life as a result of catheterization, due to complications such as vessel rupture and internal bleeding.^4^ To reduce challenges in determining FFR values, a non‐invasive CCTA‐based FFR (referred to simply as cFFR) calculation method has been proposed as passive digital twin to analyse the functional relevance of obstructive coronary lesions.^5^ This approach, through mathematical modelling and computer simulation, integrates anatomical and physiological information. Until now, the majority of the approaches that use computational modelling incorporate semi‐automated algorithms to segment the patient‐specific coronary geometry. The blood flow simulations are conducted on the extracted geometry and the boundary conditions are calculated using patients' physiological conditions. These boundary conditions are usually expressed in terms of prescribed flow rate and pressure at the proximal and distal interfaces created when isolating the vessels in the coronary network. There exist several cFFR approaches^5^, ^6^, ^7^, ^8^, ^9^, ^10^, ^11^, ^12^, ^13^, ^14^, ^15^, ^16^, ^17^, ^18^ which include the use of computationally expensive three‐dimensional models, or the use of dimensionally reduced‐order models.

**FIGURE 1:**
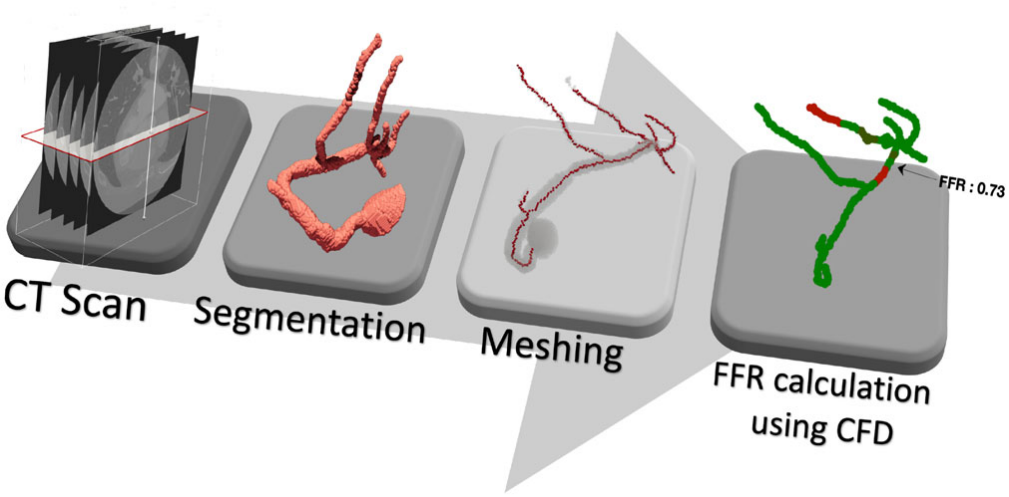
Workflow proposed in the present work for automatic calculation of FFR from a CT scan

The critical components for cFFR are, (a) extraction of geometry from Computerised Tomography (CT) scan and (b) selection of boundary conditions and computational fluid dynamics (CFD) calculations. Both coronary geometry and boundary conditions are equally important to calculate the correct cFFR. In order to obtain these components automatically, a three step process with CT scan as the input and calculated FFR as the output is presented in this work (see Figure 1). First, to identify and segment coronary arteries from CT scans, an unsupervised‐clustering based method is proposed alongside image filtration. Secondly, to extract geometrical values such as radii and centreline from the segmented geometry for mesh generation, another unsupervised method is proposed. Finally, to run blood flow simulations a coupled 1D‐0D blood flow model is employed. The Table A1 in Appendix A lists all steps and sub‐steps of the proposed automatic procedure. Following three sections respectively explain geometry extraction, meshing, CFD calculations and their integration. Section 5 provides detailed discussion on the performance of the automated cFFR calculation. Finally, Section 6 summarises the conclusions derived (Tables B2, B3, B4, B5, B6, B7, B8, B9).

## 2 GEOMETRY OF CORONARY ARTERIES

Computerised tomography (CT) scan, usually recorded in DICOM format, consists of a set of cross‐sectional images taken along a patient's longitudinal axis. These slices, usually two‐dimensional grayscale images, use regions of different grey intensities to display various internal organs and tissues (Figure 1). A form of this scan, known as CCTA is used to image the blood supply to the heart. These scans are employed in the present work to extract the geometry of the coronary arteries. The extracted arteries are used in simulating and analysing haemodynamics within them.

In the present work, data acquisition was carried out from the same site as that of Carson et al.^13^ All procedures were carried out according to standard CCTA protocols. To moderate heart rate and improve image quality during scanning, Metoprolol (beta‐blocker) was administered intravenously for some patients. The tube potential used in the scans ranged between 100 and 120 kV with prospective gating and zero padding. The prospective gating uses an electrocardiograph as a trigger to scan at a particular point in the cardiac cycle. The average in‐plane pixel spacing was 0.458 ± 0.051 mm and the slice‐spacing was 0.625 mm. Alongside CCTA data, annotations with location and value of invasively measured FFR were also provided. The CCTA data was provided in an anonymised DICOM format. The data made available is analysed in this article for coronary geometry, primarily using segmentation and lumen size estimation.

### 2.1 Segmentation

Image segmentation is a widely researched topic in computer vision and machine learning.^19^, ^20^, ^21^, ^22^ The applications of segmentation, within medical imaging, cover most parts of the human body, that is, from arteries to bones, brain to lungs and other organs.^23^ Geometries obtained from segmentation play a vital role in the diagnosis and monitoring of fatal conditions and diseases, such as malignant tumours and vascular stenoses.^24^

Methods for segmentation using traditional image processing techniques have been popular amongst researchers from the late 20th century. A number of methods using techniques such as Hessian based filtering have been widely adopted to segment coronary arteries.^25^, ^26^, ^27^, ^28^, ^29^ However, lately, the supervised learning approach using deep neural networks has gained popularity.^30^ The convolutional neural networks (CNN) hold the capacity to learn various features from images using cascading filters with non‐linear mapping. These networks are trained on vast amounts of data, thus can segment scans without pre‐processing to remove issues such as noise and leaks in the images. However, this method has two major drawbacks, (a) requirement for a vast amount of data and (b) manual segmentation and labelling during the training phase. There are methods such as transfer learning^31^ to reduce data requirement, but manual segmentation and labelling take a lot of work hours.

To reduce the time taken, an unsupervised approach for segmentation is adopted in the present work. Here, using density‐based clustering, voxels relating to the coronary arteries are clustered. Similar approaches have been proposed by Li et al^32^ and Danilov et al,^29^ with the latter having similar CPU times. However, significant variation lies in our workflow. The objective here can be divided into three parts, (a) Pre‐processing ‐ Identifying all coronary size regions. (b) Clustering ‐ Clustering of voxel centres with similar intensity that are close to each other, to form clusters of voxels. (c) Identification ‐ Identifying the correct clusters corresponding to coronary arteries. These three steps are elaborated in the following subsections.

#### 2.1.1 Pre‐processing

**FIGURE 2:**
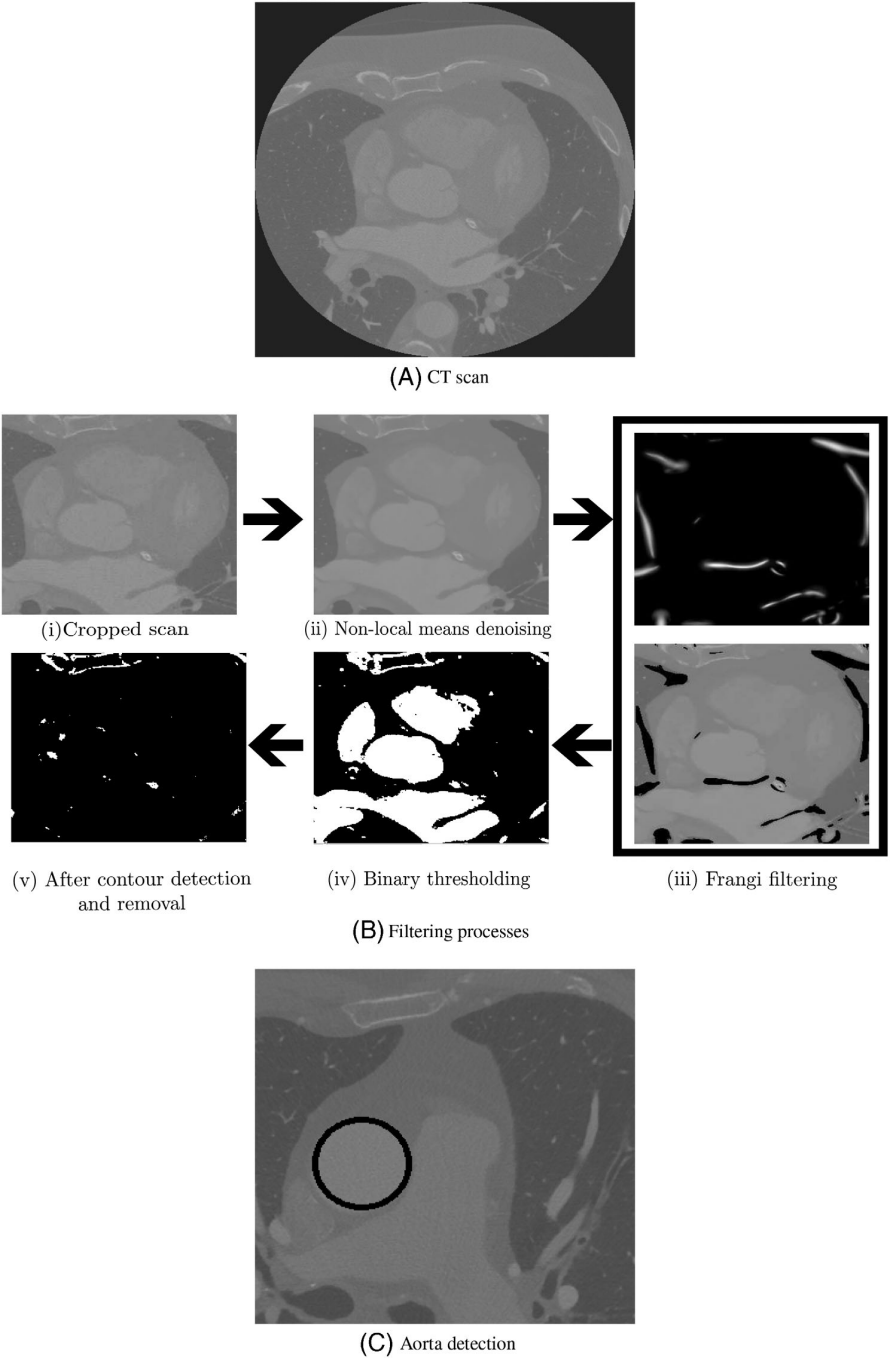
Filtering processes carried out on each of the CCTA slices to extract pixels corresponding to coronary arteries. In subfigure (B), in clockwise order, Non‐local means denoising filtering, Frangi filtering, binary thresholding, Contour detection and removal are carried out to extract parts of interest. In subfigure (C), automatic detection of aorta using Hough circle transform. The detected aorta is encircled in black

To begin with, slices of the scan are automatically cropped to focus on the cardiac region and subsequently refined with de‐noising and thresholding processes (Figure 2). This allows for unwanted artefacts to be removed from the images and convert them into binary images. For de‐noising, non‐local means method^33^ with parameters shown in Table 1 is adopted. This method removes noise from the images but preserves the different textures present in various regions, making it an optimal choice for CCTAs. The non‐local means works on the principle of finding different regions, usually disconnected, in the image that have similar grey intensities and averaging the pixel intensity within these regions.

**TABLE 1:** Filter settings used in de‐noising and Frangi filters for pre‐processing of images

Non‐local means de‐noising filter parameters		Frangi filter parameters	
Filter strength	2σ	α	0.5
Template window size	5	β	0.5
Search window size	7	γ	15

In Table 1, σ is the *SD* of Gaussian noise, calculated using wavelet based estimator from scikit‐image library.^34^, ^35^ In the present work, it is assumed that the noise in images follows a Gaussian distribution.

Following denoising, Frangi filter is used to preserve critical vessel features in the images. Frangi filter, which is a Hessian based filter, calculates eigenvectors of the Hessian matrix to compute the similarity of different regions in a given image to vessels.^36^ The values used for filter's sensitivity to deviation from a plate‐like structure, represented by α, from a blob‐like structure, represented by β, and its sensitivity to areas of high variance/texture/structures, represented by γ, are shown in Table 1. Filtered images are then subjected to global binary thresholding, in order to convert voxels with grey intensity above the selected threshold intensity value to white (255) and the rest to black (0). Following binarization, different regions in the images with white voxels are segmented using contour detection.^37^ Contours corresponding to components with an area larger than the coronary are removed using an area threshold, which is set at 900 voxels. These steps allow for voxels corresponding to components in the size range of coronary arteries to be extracted. Figure 2 shows the entire pre‐processing starting with cropping of the images.

#### 2.1.2 Clustering

Before starting the clustering process, detection of the aorta and coordinates of its centre is essential to calculate a spatial reference point. Such a reference point is later necessary to assist in the identification of voxel clusters that belong to the coronary arteries. In order to detect aorta, Hough circle transform^38^ is chosen in this work (see Figure 2). This algorithm searches for circular regions in an image having radii within a prescribed range, using edges (regions of significant local change in the image intensity), detected with the help of canny edge algorithm.^39^ The transformation is applied from the fifth slice to the ninth slice, and the mean of their aortic centre coordinates is recorded as the reference point.

For this work, minimum radius to be detected is set to 25 pixels and max radius to be detected is set at 60 pixels. These values, chosen using trial and error, allow for the detection of only one circle closer to the heart. The centre of the detected circle is used as the reference point for cluster selection in the following subsection.

The filtered white voxels of the pre‐processed image (with grey intensity of 255), need to be grouped in order to find different voxel clusters within the scan. In principle, two of these clusters will represent the left and right coronary arteries. Unsupervised method of clustering is one of the efficient and robust method to carry out such a grouping process. However, clustering works on the principle of grouping spatial points, which in the case of voxels can only be represented by the voxel centres. Therefore, voxel centres are used as the spatial points here.

To perform clustering, many existing clustering algorithms are available, however, only those approaches that preserve the geometry of vessels be selected. The K‐means, hierarchical and density based clustering, along with other variations of these methods, are a few of the widely used clustering algorithms. Amongst the three, K‐means and hierarchical clustering are ill suited for our objective. In the K‐means^40^ clustering, a number of cluster centres are pre‐selected, about which different points will be grouped iteratively. However, location for these cluster centres is usually chosen randomly. This is not preferable in our case as it allows for cluster centres to be selected outside the coronary arteries, which could lead to grouping of points that do not belong to the arteries or worse exclusion of those that actually belong to the arteries.

Further, in hierarchical clustering,^41^ nearby clusters are merged iteratively to create clusters at a higher level. The process starts with clusters of two points and then different clusters are merged iteratively based on their proximity to create clusters with increasing hierarchy. In order to get the clusters belonging to coronary, it is necessary to choose the correct hierarchy level. This selection is difficult to automate, especially if the scan contains discontinuities.

**FIGURE 3:**
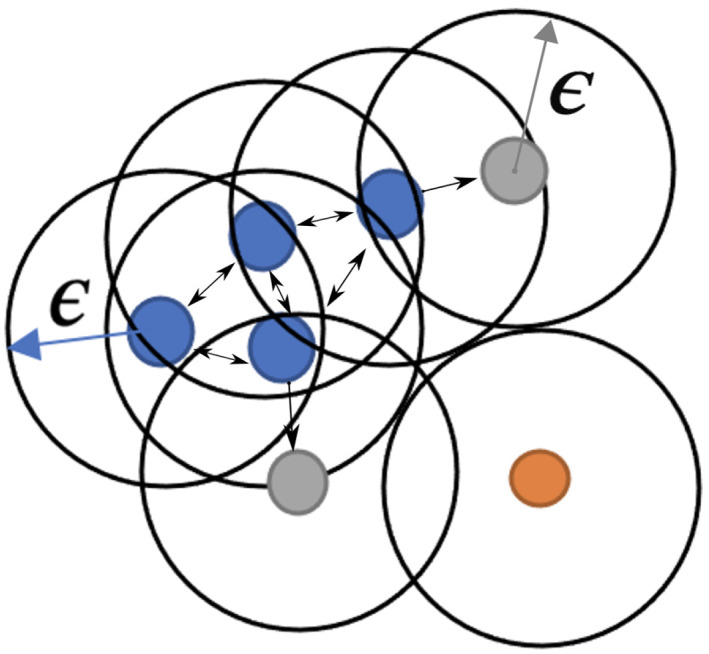
Density‐based spatial clustering of applications with noise (DBSCAN). Points within each other's vicinity, with a search radius of ε are grouped together as a single cluster. Blue and grey points belong to one cluster, where the former ones are core points. The grey points are reachable but make up the outer points of the cluster. The orange point is a noise point as it cannot be reached by any other point

Thus, in the present work, a density‐based spatial clustering of applications with noise (DBSCAN),^42^ is adopted. This algorithm identifies clusters in an array of points based on their density in a given spatial region. Since points, representing centres of white voxels, corresponding to coronary arteries are densely packed, this algorithm is optimal to cluster them. Amongst the many clusters that emerge after DBSCAN clustering, two clusters will, in principle, represent the coronary geometry. In this algorithm, as shown in Figure 3, points that lie within each other's preset search radius, represented by ε, and also have a preset minimum number of points within this search area are grouped together (blue and grey points). Within such a group, points with more than preset minimum neighbouring points form the core points (blue points) and those which are reachable by the core points but have no further neighbouring points are considered outer points (grey points). Further, points with less than the preset minimum number of neighbouring points in the search area are considered as noise points (orange point).

For clustering of points belonging to the coronary arteries, ε, search radius, is chosen as 1.6 voxels and minimum points needed within the search area is chosen as 2.

#### 2.1.3 Cluster identification

**FIGURE 4:**
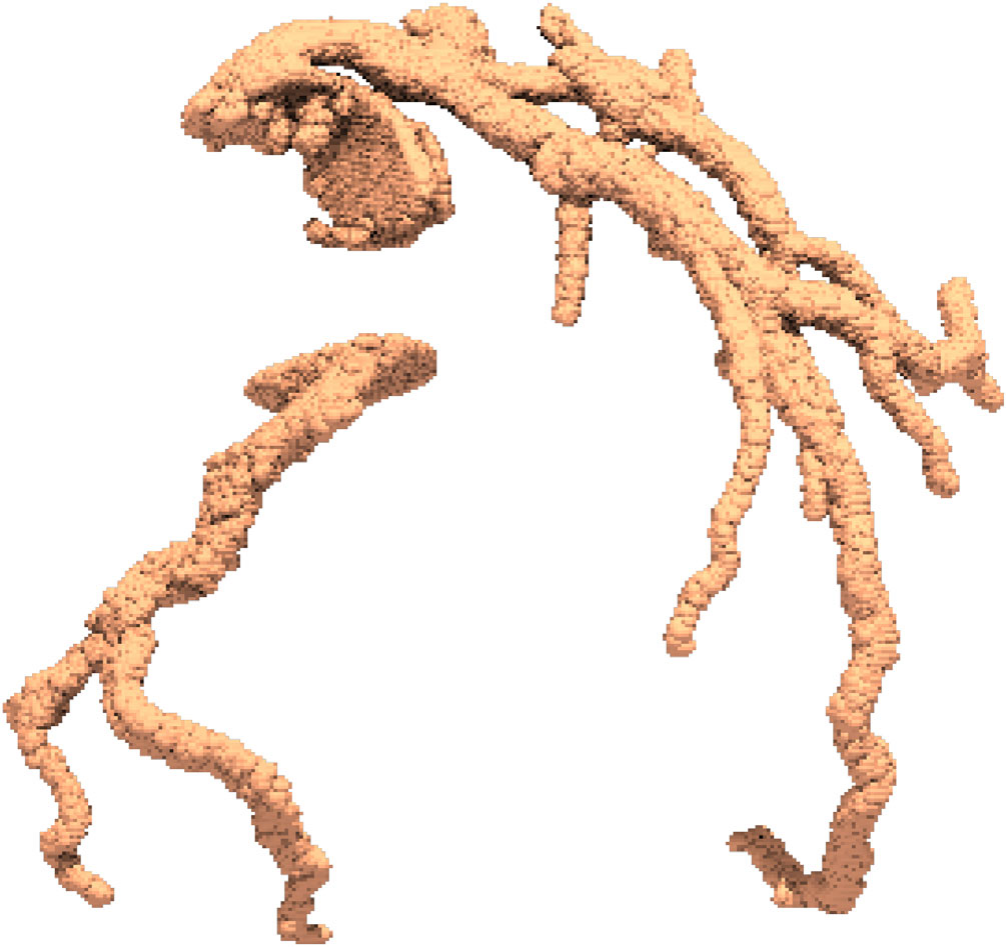
Detected clusters of voxels of coronary arteries. The left cluster and right cluster are of right and left coronary arteries respectively

After clustering, various clusters of voxels (represented by their centres) emerge. However, only two of these clusters are the coronary arteries. In order to make our process automatic, two clusters corresponding to the left and right coronary arteries are chosen based on their proximity and orientation to the aorta (see Figure 4). By adopting this approach we eliminate the chances of incorrect cluster labelling, especially to avoid vessels in the pulmonary region, which sometimes can have geometry similar to that of the coronary arteries. Out of all clusters detected in Section 2.1.2, two clusters with points having least distance to the aortic centre, a spatial reference point identified in Section 2.1.2, are chosen as the coronary clusters.

## 3 ESTIMATION OF LUMEN SIZE FOR MESH GENERATION

The voxel volume corresponding to coronary arteries, extracted from the above processes, is studied here for extracting geometrical values of the lumen. The coronary geometry is extracted using a combination of skeletonisation and surface meshing. However, before extracting the geometry, voxel volume is filtered to remove vessels smaller than a fixed size and also large volumes belonging to aortic root, thereby focusing on coronary vessels. To perform this DBSCAN is used. The density based clustering using DBSCAN was previously employed to detect coronary voxel volume from the filtered images. This algorithm is used again, however this time only on the selected voxel volume, with a setting of 7 voxel search radius,ε, and a minimum 700 points in this radius to detect and remove voxels from aortic root and a setting of 1.5 voxel search radius and a minimum 3 points in this radius to remove small vessels.

**FIGURE 5:**
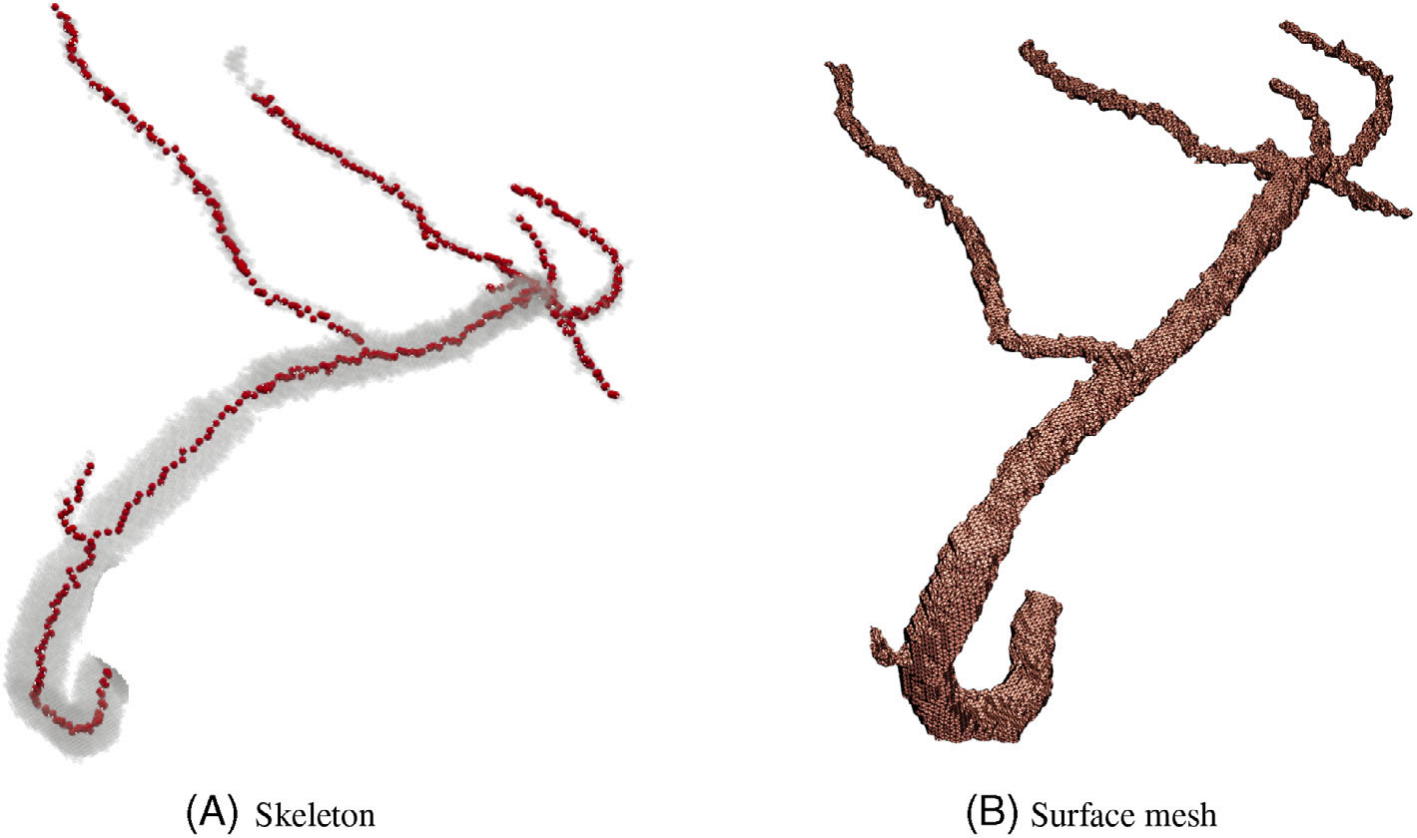
Skeleton obtained from voxel cluster is used as centreline along which lumen radii is calculated

The skeletonisation algorithm, adopted from Scikit image,^34^ originally proposed by Lee et al,^43^ uses the logic of removing boundary voxels in order to thin down a volume of voxels until a middle voxel along the vessel axis is left out. These thinned down voxels make up the skeleton of vascular volume and act as the centreline for mesh generation(see Figure 5). The obtained centreline, which usually is a tree representing the coronary artery network, is split into individual branches for radii estimation in each of them. To split the skeleton into individual vessel branches, Skeleton network from ImagePy library^44^, ^45^ is used.

In order to extract radius from the volume, a surface Mesh is generated using marching cube algorithm^46^(see Figure 5). This popular algorithm utilises a traversing cube, in a volume discretised into cubes, for extracting a polygonal mesh of an iso‐surface from voxels.

The cross‐section of blood vessels are assumed to be circular for modelling purposes as explained in Section 4. Thus, an approximate circular radii needs to be calculated along the centreline. The vertices on the surface mesh are utilised to calculate such a vessel radius from the centre line. In the present work, the shortest normal distance between a point on the centreline to the wall vertices is chosen as the radius for that given point.

Finally, the points on the centreline and radii corresponding to them are interpolated using PCHIP, Piecewise Cubic Hermite Interpolating Polynomial, to generate an approximately uniform one‐dimensional mesh along the vessel's centreline.

## 4 HAEMODYNAMIC MODELLING

The coronary lumen geometry extracted from CT scan is used to analyse blood flow using a coupled 1D‐0D model. The one dimensional model, formulated using Equations (1) and (2), is used to analyse vascular flow with the help of lumped models, to represent downstream resistance from the microvasculature. The continuity and momentum equations are:

(1) $$C_{A}\frac{\partial P}{\partial t}+\frac{\partial Q}{\partial x}=0,$$

and

(2) $$\frac{\rho }{A}\frac{\partial Q}{\partial t}+\frac{\rho }{A}\frac{\partial \left(Q^{2}/A\right)}{\partial x}+\frac{\partial P}{\partial x}=-\frac{22\mathrm{\mu \pi Q}}{A^{2}}$$

where $C_{A}$ is the compliance, P is the hydrostatic pressure, Q is the volumetric flow rate, A is the cross‐sectional area *ρ* = 1.06 g/cm^3^ is the density of blood, and μ=0.04P (Poise) is the dynamic viscosity, t and x are the temporal and spatial coordinates, respectively. The viscous friction term on the right side of the momentum equation is responsible for predicting the pressure drop due to the vessel narrowing. The second term on the left side of this equation is also important for predicting the pressure drop, particularly if the area before and after a stenosis is different. A fine spatial mesh of 0.1 mm is required to accurately account for sudden changes in geometry. A non‐linear visco‐elastic constitutive law (Equation 3),^47^ is used to complete the system.

(3) $$P-P_{\mathrm{ext}}-P_{0}=\frac{2\rho c_{0}^{2}}{b}\left({\left(\frac{A}{A_{0}}\right)}^{b/2}-1\right)+\frac{\Gamma }{A_{0}\sqrt{A_{0}}}\frac{\partial A}{\partial t}$$

where $P_{\mathrm{ext}}$ is the external pressure, $P_{0}$ is a reference pressure, $A_{0}$ is the cross‐sectional area at the reference pressure, and b is:

(4) $$b=\frac{2\rho c_{0}^{2}}{P_{0}-P_{\text{collapse}}}$$

with $P_{\text{collapse}}$, collapsing pressure, and $c_{0}$, reference wave speed of the vessel, which is calculated as:

(5) $$c_{0}=\sqrt{\frac{2}{3\rho }\left(k_{1}\exp\left(k_{2}r_{0}\right)+k_{3}\right)}$$

with *k*_1_ = 2 × 10^7^ g^2^/cm/s, *k*_2_ = –22.53 cm^–1^, *k*_1_ = 8.65 × 10^5^ g^2^/cm/s, and $r_{0}$ being the reference radius of the vessel.

**FIGURE 6:**
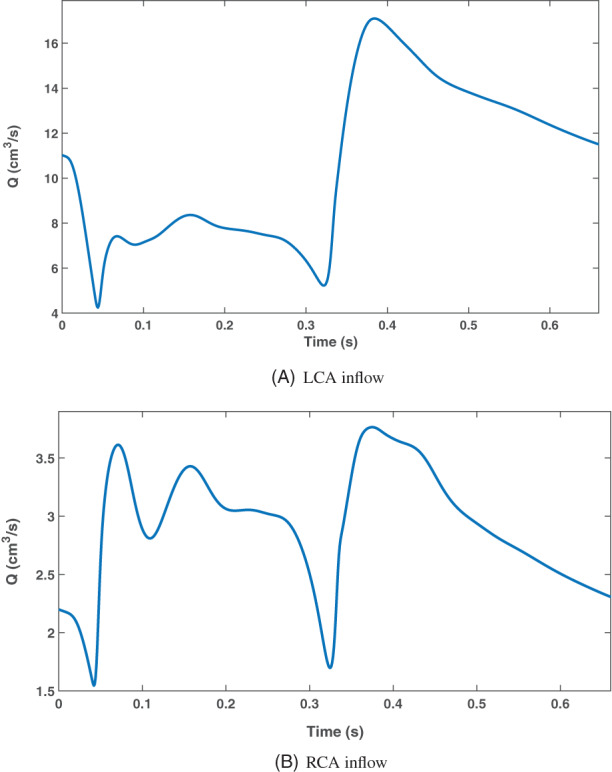
Inflow boundary conditions used for left and right coronary arteries

The boundary conditions, inlet conditions and ventricular pressures, for left and right coronary arteries are created using a closed‐loop model. However, the boundary conditions are chosen to be the same for all patients, as we have no additional patient information other than the CT data. The inflow rate of the left and right coronary arteries are shown in Figure 6. Due to this lack of patient data, coronary dominance was not considered in this modelling approach.

**FIGURE 7:**
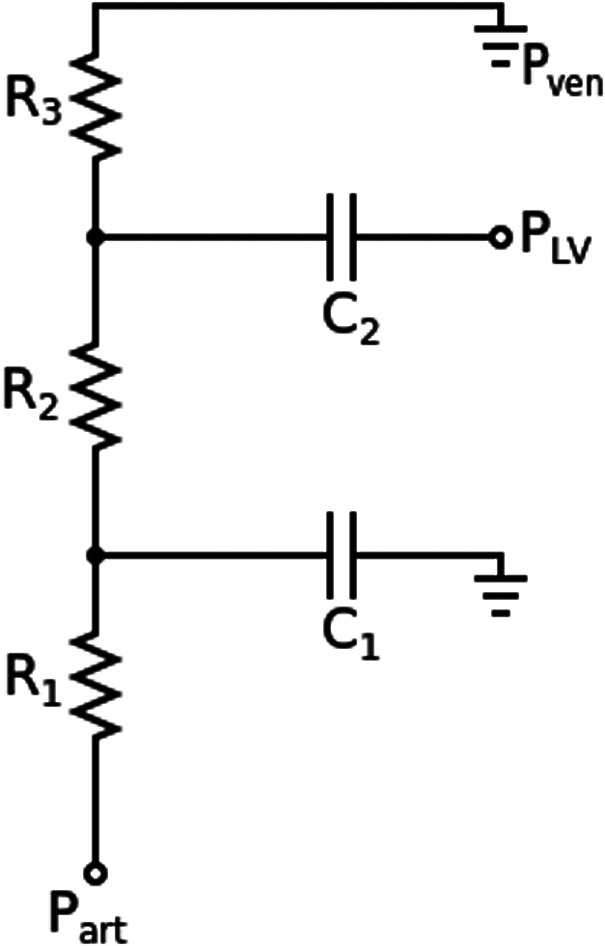
A lumped‐parameter model connected to the outlets of the patient specific coronary network to represent the micro‐circulation. Part connects to the 1D domain,$R_{1}$ is the characteristic impedance, $R_{2}$ is the resistance of the micro‐circulation at the arterial side, $R_{3}$ is the micro‐circulatory resistance at the venous side, $C_{1}$ is the micro‐circulatory arterial compliance, $C_{2}$ is the intra‐myocardial compliance, $P_{\mathrm{LV}}$ is a scaled pressure from the left ventricle (or right ventricle for the right coronary artery [RCA]), and $P_{\mathrm{ven}}$ represents the pressure in the venous system which is set to 5 mmHg

The coronary artery resistance is calculated as:

(6) $$R_{\mathrm{cor},i}=\frac{\mathrm{MAP}}{Q_{\mathrm{cor},i}}$$

where MAP is a weighted average of an idealised diastolic and systolic pressure and $Q_{\mathrm{cor},i}$ is the inflow rate in the left (or right) coronary artery. The weighted average for MAP is $\frac{2}{3}\times$ diastolic pressure $+\frac{1}{3}\times$ systolic pressure, where diastolic pressure is 80 mmHg, and systolic pressure is 120 mmHg. The distribution of resistance throughout each branch is determined using a variant of Murray's power law, with a power of 2.27 as in van der Giessen et al,^48^ with vascular bed compliance distributed in a similar way.^49^ The coronary vascular bed model is shown in Figure 7, which includes an external pressure acting from the heart ventricles. In Figure Windkessel, the parameters of lumped‐parameter model for each coronary vascular bed is calculated as $R_{1}=\rho \frac{c_{0}}{A_{0,\mathrm{end}}}$, $R_{2}=0.79\times \left(R_{\mathrm{Tf}}-R_{1}\right)$, $R_{3}=0.21\times \left(R_{\mathrm{Tf}}-R_{1}\right)$, where $A_{0,\mathrm{end}}$ is the area at the end of the terminal vessel to which the vascular bed is connected and $R_{\mathrm{Tf}}$ is the resistance corresponding to the terminal vessel's fraction of total coronary resistance as per the distribution of resistance calculated using Murray's power law.

In Figure 7, a lumped‐parameter model is shown as an electric circuit due to the analogous nature of its components with hydrodynamics. $R_{1}$ is the characteristic impedance, $R_{2}$ is the resistance of the micro‐circulation at the arterial side, $R_{3}$ is the micro‐circulatory resistance at the venous side, $C_{1}$ is the micro‐circulatory arterial compliance, $C_{2}$ is the intra‐myocardial compliance. The compliances are given by,

(7) $$Q_{\mathrm{net}}=C\frac{\partial P_{\mathrm{tm}}}{\partial t},$$

where $P_{\mathrm{tm}}$ is the transmural pressure, difference between pressure inside the vessel to pressure outside the vessel (from the surrounding tissue), and t is the temporal coordinate. Various other lumped‐parameter models have been proposed by different publications with more detailed modelling of vascular beds and can be found in the references.^16^, ^17^, ^18^ The full 1D‐0D system is solved implicitly using a sub‐domain collocation scheme referred to as the enhanced trapezoidal rule method.^13^, ^50^ The scheme uses a second‐order backward difference temporal discretisation, and a composite trapezoidal rule for the spatial discretisation of the 1D domain. The steps taken in this section for haemodynamic modelling of coronary geometries have been given in Table 2.

**TABLE 2:** A summary of steps followed in this article for haemodynamic modelling of the coronary artery geometries obtained using the proposed automatic method

Haemodynamic modelling of the coronary artery geometries obtained using the proposed automatic method
Import 1D mesh (centerline with equidistant nodes and vessel radii data at each of these nodes)
Set boundary and initial conditions → Input inlet boundary conditions (as shown in Figure 6, calculated using closed‐loop model) → Calculate total resistance → As per Murray's power law: – Distribute resistance throughout each branch. – Distribute coronary vascular bed compliance → Calculate parameters for lumped‐parameter models at each terminal vessel
Solve full 1D‐0D system using enhanced trapezoidal rule method
Calculate cFFR using blood pressure values obtained.

## 5 RESULTS AND DISCUSSIONS

The quintessence of methodology presented in this work is to process every step from CCTA scan to final cFFR value calculation without any manual intervention. Twenty five CCTAs, each belonging to different patients, were chosen to test the proposed automatic methodology. This section analyses the performance of the proposed workflow in terms of accuracy of segmented coronary geometry and the cFFR results obtained for the test patient cohort. Limitations to the degree of automation and their potential solutions have also been discussed in this section.

Segmentation accuracy is one of the primary factors that will affect the accuracy of cFFR calculated using the proposed workflow. In order to estimate this accuracy, comparison with manually segmented coronary geometry is carried out for the CCTAs from the test patient cohort. The manual segmentation was carried out by the second author^13^ using widely available Vascular Modelling toolkit (VMTK) software. Table 3, summarises the results obtained from this geometry comparison, however, a detailed comparison for each case from the test patient cohort is made available to the readers in Appendix B. In Table 3, it can be observed that the automatically segmented coronary geometry, using the proposed workflow, is similar to that of manually segmented geometry. The average vessel length (L), radii at the start of the vessel ($R_{0}$) and radii at the end of the vessel ($R_{f}$) of both left (LCA) and right (RCA) coronary arteries obtained automatically are similar or close to that of manually segmented geometry. However, the average length and final vessel radius obtained for the left circumflex artery (LCX) and left anterior descending artery (LAD) using the proposed workflow is higher than that of manual segmentation, providing an observation that automatic workflow can detect narrower vessels with ease. Further, the similarity in minimum vessel radius at the stenosis ($R_{s}$), provides confidence in geometry obtained automatically from CCTAs.

**TABLE 3:** Coronary geometry detected from the proposed automatic segmentation workflow is compared against manually segmented coronary arteries. L, $R_{0}$ and $R_{f}$, are average vessel length, radius at the start of the vessel and radius at the end of the vessel respectively. $R_{s}$ is the average minimum vessel radius at the stenosis location. Detailed data for each patient case is available in Appendix B

	LCA		LAD		LCX		RCA	
	Automatic	Manual	Automatic	Manual	Automatic	Manual	Automatic	Manual
L (cm)	1.00 (*SD*: 0.50)	0.76 (*SD*: 0.54)	8.71 (*SD*: 2.75)	6.95 (*SD*: 2.60)	7.51 (*SD*: 3.02)	4.56 (*SD*: 2.13)	9.98 (*SD*: 2.58)	12.09 (*SD*: 2.26)
$R_{0}$ (mm)	1.60 (*SD*: 0.35)	1.63 (*SD*: 0.47)	1.38 (*SD*: 0.31)	1.40 (*SD*: 0.33)	1.35 (*SD*: 0.34)	1.35 (*SD*: 0.30)	1.52 (*SD*: 0.41)	1.57 (*SD*: 0.29)
$R_{f}$ (mm)	1.55 (*SD*: 0.45)	1.53 (*SD*: 0.29)	0.66 (*SD*: 0.29)	0.88 (*SD*: 0.16)	0.69 (*SD*: 0.27)	1.03 (*SD*: 0.40)	0.96 (*SD*: 0.56)	0.92 (*SD*: 0.20)

$R_{s}$(mm)			
Left		Right	
Automatic	Manual	Automatic	Manual
0.68 (*SD*: 0.30)	0.67 (*SD*: 0.21)	0.84 (*SD*: 0.50)	0.70 (*SD*: 0.21)

**FIGURE 8:**
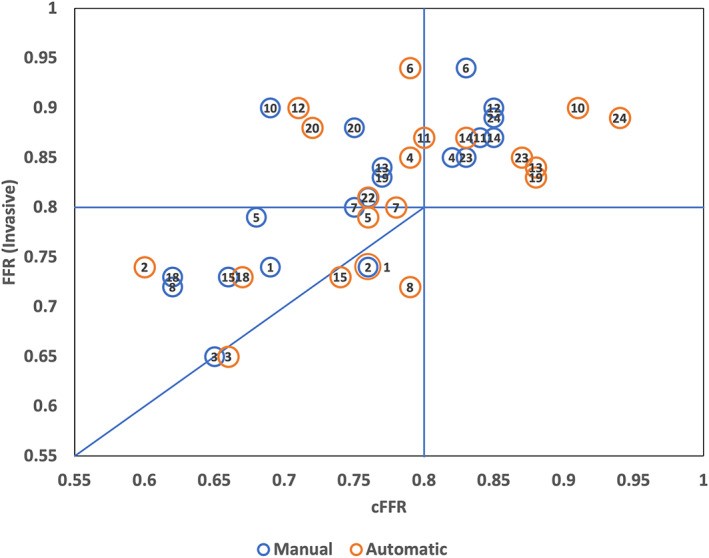
cFFR values obtained from models using automatically and manually segmented coronary geometries are compared against invasively measured FFR. The cases for which invasive FFR values were not available have been excluded from the graph to avoid any confusion; however, cFFR values for such cases are available in Table 4

In Table 4 and Figure 8, the cFFR values obtained are shown. In most of the cases, the cFFR value calculated are observed to be close to that of actual measured FFR, which is invasive in nature.

**TABLE 4:** cFFR values calculated using geometries obtained automatically and manually on test patient cohort is compared against actual invasively measured FFR

	Location	cFFR(automatic)	cFFR (manual)	FFR(invasive)
Patient 1	Left	0.76	0.69	0.74
Patient 2	Left	0.60	0.76	0.74
Patient 3	Left	0.66	0.65	0.65
Patient 4	Left	0.79	0.82	0.85
Patient 5	Left	0.76	0.68	0.79
Patient 6	Right	0.79	0.83	0.94
Patient 7	Left	0.78	0.75	0.80
Patient 8	Left	0.79	0.62	0.72
Patient 9	Left	0.73	0.78	NA
Patient 10	Left	0.91	0.69	0.90
Patient 11	Left	0.80	0.84	0.87
Patient 12	Left	0.71	0.85	0.90
Patient 13	Left	0.88	0.77	0.84
Patient 14	Right	0.83	0.85	0.87
Patient 15	Left	0.74	0.66	0.73
Patient 16	Left	0.58	0.62	NA
Patient 17	Left	0.75	0.77	NA
Patient 18	Left	0.67	0.62	0.73
Patient 19	Left	0.88	0.77	0.83
Patient 20	Left	0.72	0.75	0.88
Patient 21	Left	0.72	0.68	NA
Patient 22	Left	0.76	0.76	0.81
Patient 23	Right	0.87	0.83	0.85
Patient 24	Right	0.94	0.85	0.89
Patient 25	Left	0.51	0.42	NA
		Mean: 0.76 (*SD*: ±0.10)	Mean: 0.73 (*SD*: ±0.10)	Mean: 0.82 (*SD*: ±0.077)

**FIGURE 9:**
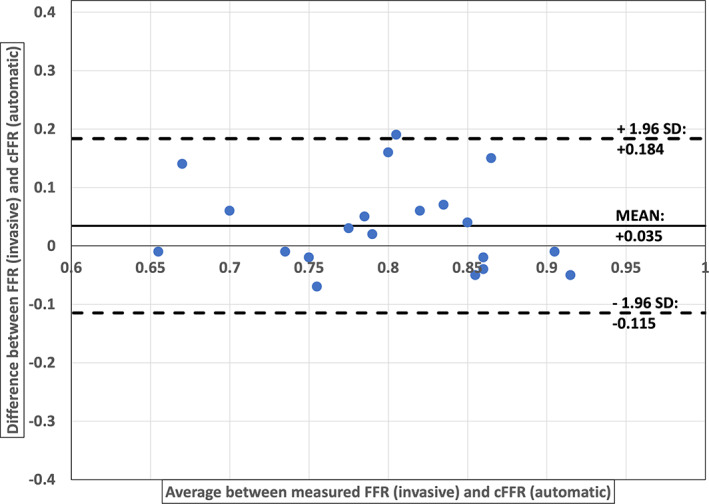
Band Altman plot to analyse the agreement between FFR (invasive) and cFFR (automatic) values from Table 4

Though most of the results are in an acceptable range, a significant difference in patients 2, 6, and 12 can be observed. This could be attributed to either poor scan quality or incorrect selection of boundary conditions. It must be recollected that fixed input boundary conditions for left and right coronary arteries are used in this work owing to the lack of patient details. Fixed input boundary conditions can lead to incorrect calculation of cFFR, especially in patients with co‐morbidities such as hypertension, aortic stenosis and cardiomyopathy, as crucial parameters affecting the patient's outflow which in turn affects coronary flow gets ignored. In general, an underestimation of cFFR values compared to invasively measured FFR is observed, particularly during manual segmentation. This may be attributed to the fact that radii from manual segmentation are slightly lower as voxels near the lumen wall are more efficiently captured during automatic than manual segmentation. In the automatic segmentation, multiple grey intensity thresholds are used during processing (explained in Section 5.1), which allows for voxels near the boundary layer that have a low intensity to be captured. However, if a threshold of 0.8 is assumed as the critical value, none of the results has any false negatives. Thus, the proposed workflow produced satisfactory results, providing confidence towards using such a system in clinical environments (Figure 9).

### 5.1 Limitations

Proposed automatic method gives satisfactory cFFR values in most cases, however, in a small number of cases (20%), the automatic method either failed to produce or produced erroneous cFFR values. In all such cases, minor changes to the configuration, such as changes in filter settings, were observed to resolve any breakdown and satisfactory cFFR values were calculated without any further manual intervention. Since only minor adjustments to parameters were sufficient to resolve any problems, confidence is established in the core working principle of the proposed methodology. Majority of breakdowns in the automatic process occurred when analysis was carried out on scans of poor quality. Such scans usually had too many holes in them. In the process of making minor adjustments, the following challenges and their possible solutions were identified in the system.

During segmentation, binary thresholding is vital for identifying all voxels of interest so as to obtain their co‐ordinates for clustering. The selection of the grey intensity threshold value is extremely vital for extraction of correct and complete vascular geometries. Various factors such as dye concentration, blood composition, calcification and pre‐existing stents can affect the grey intensity in the scans. The intensity also decreases along the downstream direction of an artery. Since the orientation of arteries and patient parameters vary drastically, intensity‐based thresholding methods in some cases either fail completely or partially in capturing the geometry. To alleviate this, 5–7 copies of the CCTA were simultaneously filtered and clustered individually. Each of these individual copies used a different grey intensity threshold varying within the range of 125–145. Upon completion, voxels from each individual copy classified as that of belonging to the coronary arteries, were combined. This approach, however, wasn't efficient in scans with discontinuities or inaccurate data. Since density based scanning identifies neighbouring points(voxel centres) using a search radius, incorrect voxels(from discontinuities) within this search area lead to major leaks. Interestingly, it was observed that most of the leaks occurred in regions closer to the aortic root. Manual intervention was required in order to select only those individual copies that had good quality cluster, which consequently was merged and analysed upon automatically.

The other issue faced was during cleaning/filtering of arterial voxel volume after clustering to remove small vessels. In some geometries, due to a presence of occlusion (atherosclerosis), lumen had cross‐sectional area with very small voxel volume. These regions had a voxel density lower than the threshold values preset to remove small vessels, leading to loss of vessel geometry around the occlusion (atherosclerosis). This was undesirable as lowering the density threshold value in order to preserve geometry would allow for smaller vessels to be added to the mesh. Such geometry would reduce the performance of one dimensional code by delaying convergence and affecting approximations. In the two cases where this issue was observed, the density threshold had to be decreased and the smaller vessels had to be ignored during haemodynamic modelling.

The final issue faced was the deletion of initial few nodes in either of the coronary arteries. The section of coronary artery emerging from the aortic root is affected during filtering of coronary volume. This removal of geometry, belonging to aortic root, in some cases removed voxels disproportionately from the initial lumen region of the coronary arteries. This lead to smaller diameter being calculated for nodes in such regions. In turn it affected the complete cFFR calculation downstream. To alleviate this issue, first 4–6 nodes were not considered during haemodynamic analysis.

For future work, breakdowns like these can be avoided by training a supervised monitoring system. Such a system could intervene and adjust parameters if any of the above observed problems arise. A simple closed loop system or neural networks can be used for such control.

## 6 CONCLUSIONS

The proposed methodology, built on a combination of unsupervised learning and CFD, provides a robust platform to automatically calculate cFFR values. Satisfactory results observed by testing it on a patient cohort of 25 patients provides the required assurance that the method is reliable. Thus, it can be concluded that automating the process of calculating cFFR from CT scans is feasible and reliable. The entire workflow presented in this article take only between 12 and 25 min per patient. Thus, the automated method proposed is rapid and suitable for fast functional assessment of arteries.

In addition, it is worth noting that even though the working principle fundamentally varies from the popular rising trend of using supervised neural networks, present work provides a potential for future combination of such methodologies to enhance accuracy of cFFR calculation and computational performance.

## Data Availability

The data that support the findings of this study are available on request from the corresponding author. The data are not publicly available due to privacy or ethical restrictions.

## Appendix A Workflow summary

**TABLE A1:** Workflow proposed in the present work to automate cFFR calculation from CT scans

Workflow proposed in the present work to automate cFFR calculation from CT scans
**Segmentation** ▴ Pre‐processing (to identify regions having area similar to the coronary arteries) → Denoising (using non‐local means algorithm) → Frangi filtering → Binary thresholding → Contour detection and removal ▴ Clustering → Aorta detection (using Hough circle transform) → Density based clustering of white voxels (using DBSCAN algorithm) ▴ Identification of cluster **Estimation of lumen size** → Skeletonisation (to obtain the centerline) → Surface mesh generation (using marching cube algorithm) → Splitting of centreline into individual vessels → Radii calculation along the centreline → 1D mesh generation (using centreline and radii along it) **Computational Fluid Dynamics** (to calculate cFFR using a 1D‐0D blood flow model) → Import 1D mesh → Set boundary and initial conditions: ⋄ Input inlet boundary conditions (as shown in Figure 6, calculated using closed‐loop model) ⋄ Calculate total resistance ⋄ As per Murray's power law: → Distribute resistance throughout each branch. → Distribute coronary vascular bed compliance ⋄ Calculate parameters for lumped‐parameter models at each terminal vessel ⋄ Set initial boundary conditions → Solve full 1D‐0D system using enhanced trapezoidal rule method → Calculate cFFR using blood pressure values obtained

## Appendix B Vessel geometry of coronary arteries with stenosis

**TABLE B2:** Values obtained from the proposed workflow for left coronary geometry is compared against values obtained from manual segmentation carried out using Vascular Modelling toolkit (VMTK)

	Length								
	LCA			LAD			LCX		
	Automatic	Manual	RE (×100)	Automatic	Manual	RE (×100)	Automatic	Manual	RE (×100)
Patient 1	1.46	0.91	0.60	11.12	5.1	1.18	3.88	2.06	0.88
Patient 2	0.89	0.9	0.01	7.23	9.53	0.24	4.03	1.31	2.08
Patient 3	1.51	0.63	1.40	9.34	9.05	0.03	7.08	6.96	0.02
Patient 4	1.12	1.04	0.08	4.93	13.3	0.63	7.15	4.7	0.52
Patient 5	1.01	0.34	1.97	4	4.28	0.07	6.5	4.67	0.39
Patient 7	0.21	0.63	0.67	8.58	6.11	0.40	10.69	3.42	2.13
Patient 8	0.88	2.12	0.58	5.33	7.56	0.29	2.57	4.89	0.47
Patient 9	0.51	0.58	0.12	11.17	9.39	0.19	12.11	3.43	2.53
Patient 10	1.35	1.98	0.32	10.42	8.47	0.23	10.22	3.76	1.72
Patient 11	0.81	0.96	0.16	10.7	7.608	0.41	12.17	9.61	0.27
Patient 12	1.41	0.67	1.10	9.78	1.01	8.68	6.71	6.24	0.08
Patient 13	0.75	0.41	0.83	5.56	5.61	0.01	7.16	2.07	2.46
Patient 15	1.2	0.2	5.00	8.96	4.18	1.14	5.6	4.27	0.31
Patient 16	0.97	0.4	1.43	13.21	6.58	1.01	11.63	6.23	0.87
Patient 17	0.67	0.46	0.46	3.6	8.58	0.58	6.37	2.21	1.88
Patient 18	0.51	1.01	0.50	11.61	7.53	0.54	9.74	6.34	0.54
Patient 19	2.42	1.49	0.62	8.31	6.07	0.37	2.35	1.34	0.75
Patient 20	0.54	0.33	0.64	8.76	4.28	1.05	8.98	4.03	1.23
Patient 21	0.37	0.32	0.16	10.16	5.09	1.00	6.24	5.77	0.08
Patient 22	1.53	0.43	2.56	12.28	8.97	0.37	5.94	6.83	0.13
Patient 25	0.91	0.26	2.50	7.94	7.7	0.03	10.67	5.62	0.90

**TABLE B3:** Initial vessel radius obtained from the proposed workflow for left coronary geometry is compared against values obtained from manual segmentation carried out using Vascular Modelling toolkit (VMTK)

	$R_{0}$								
	LCA			LAD			LCX		
	Automatic	Manual	RE (×100)	Automatic	Manual	RE (×100)	Automatic	Manual	RE (×100)
Patient 1	1.51	1.46	0.03	1.21	1.01	0.20	1.03	1.19	0.13
Patient 2	1.8	1.73	0.04	1.31	1.2	0.09	1.07	1.41	0.24
Patient 3	1.24	1.38	0.10	1.1	1.41	0.22	1.3	1.42	0.08
Patient 4	2.31	1.97	0.17	1.79	2.3	0.22	1.89	2.13	0.11
Patient 5	1.23	1.04	0.18	0.77	1.08	0.29	0.75	1.22	0.39
Patient 7	1.4	1.34	0.04	1.3	1.39	0.06	1.44	1.34	0.07
Patient 8	1.96	1.59	0.23	1.46	1.83	0.20	1.63	1.57	0.04
Patient 9	1.96	2.01	0.02	1.6	1.45	0.10	1.65	1.36	0.21
Patient 10	1.6	3.22	0.50	1.14	1.69	0.33	0.82	1.98	0.59
Patient 11	1.88	2.17	0.13	1.61	1.5	0.07	1.38	1.43	0.03
Patient 12	1.87	1.51	0.24	1.69	1.32	0.28	1.71	1.26	0.36
Patient 13	1.64	1.61	0.02	1.65	0.94	0.76	1.64	0.93	0.76
Patient 15	1.71	1.99	0.14	1.42	1.53	0.07	1.13	1.38	0.18
Patient 16	1.39	1.09	0.28	1.2	1.43	0.16	1.18	0.91	0.30
Patient 17	1.49	1.38	0.08	1.7	1.47	0.16	1.88	1.4	0.34
Patient 18	1	1.18	0.15	1.8	1.24	0.45	1.71	1.36	0.26
Patient 19	1.71	1.68	0.02	1.67	1.32	0.27	1.12	1.02	0.10
Patient 20	1.09	1.33	0.18	0.79	1.02	0.23	1.06	1.22	0.13
Patient 21	1.2	1.59	0.25	0.95	1.26	0.25	1.29	1.28	0.01
Patient 22	2.1	1.55	0.35	1.44	1.88	0.23	1.6	1.56	0.03
Patient 25	1.51	1.46	0.03	1.46	1.03	0.42	1.13	0.95	0.19

**TABLE B4:** Final vessel radius obtained from the proposed workflow for left coronary geometry is compared against values obtained from manual segmentation carried out using Vascular Modelling toolkit (VMTK)

	$R_{f}$								
	LCA			LAD			LCX		
	Automatic	Manual	RE (×100)	Automatic	Manual	RE (×100)	Automatic	Manual	RE (×100)
Patient 1	1.61	1.21	0.33	0.54	0.99	0.45	0.96	1.02	0.06
Patient 2	1.6	1.37	0.17	0.63	1	0.37	0.6	0.98	0.39
Patient 3	1.33	1.42	0.06	0.83	0.76	0.09	0.85	1.15	0.26
Patient 4	2.68	2.25	0.19	1.36	0.98	0.39	1.15	1.03	0.12
Patient 5	0.94	1.64	0.43	0.53	0.96	0.45	0.34	0.77	0.56
Patient 7	1.33	1.53	0.13	0.62	0.99	0.37	0.53	1.24	0.57
Patient 8	1.63	1.8	0.09	0.83	0.96	0.14	0.62	1.32	0.53
Patient 9	2.07	2.01	0.03	0.51	0.55	0.07	0.97	1.1	0.12
Patient 10	1.33	1.69	0.21	0.34	0.67	0.49	0.26	0.87	0.70
Patient 11	1.88	1.26	0.49	0.27	0.71	0.62	0.46	0.85	0.46
Patient 12	0.9	1.46	0.38	0.6	1.24	0.52	0.84	0.7	0.20
Patient 13	1.51	1.49	0.01	1.25	0.95	0.32	0.36	0.67	0.46
Patient 15	1.72	1.7	0.01	0.85	0.83	0.02	0.72	0.98	0.27
Patient 16	1.41	1.11	0.27	0.5	0.85	0.41	0.88	0.91	0.03
Patient 17	1.61	1.59	0.01	0.48	0.9	0.47	0.67	1.2	0.44
Patient 18	1.17	1.45	0.19	0.44	0.78	0.44	0.84	0.88	0.05
Patient 19	1.8	1.59	0.13	0.46	0.97	0.53	1.02	0.89	0.15
Patient 20	1.04	1.3	0.20	0.45	0.69	0.35	0.24	0.61	0.61
Patient 21	1.38	1.05	0.31	0.47	0.83	0.43	0.92	0.85	0.08
Patient 22	2.46	1.72	0.43	1.1	1.07	0.03	0.78	2.59	0.70
Patient 25	1.2	1.4	0.14	0.87	0.83	0.05	0.41	1	0.59

**TABLE B5:** Minimum vessel radius at stenosis location obtained from the proposed workflow for left coronary geometry is compared against values obtained from manual segmentation carried out using Vascular Modelling toolkit (VMTK)

	$R_{s}$		
	LCA		
	Automatic	Manual	RE (×100)
Patient 1	0.42	0.91	0.54
Patient 2	0.72	0.69	0.04
Patient 3	0.63	0.56	0.13
Patient 4	0.91	0.63	0.44
Patient 5	0.52	0.64	0.19
Patient 7	0.64	0.61	0.05
Patient 8	1.57	0.64	1.45
Patient 9	0.43	0.65	0.34
Patient 10	0.62	0.69	0.10
Patient 11	0.36	0.69	0.48
Patient 12	0.57	0.53	0.08
Patient 13	0.76	0.55	0.38
Patient 15	1	1.15	0.13
Patient 16	0.32	0.61	0.48
Patient 17	0.48	0.61	0.21
Patient 18	1.15	0.61	0.89
Patient 19	0.96	0.87	0.10
Patient 20	0.31	0.58	0.47
Patient 21	0.71	0.56	0.27
Patient 22	0.59	0.94	0.37
Patient 25	0.65	0.41	0.59

**TABLE B6:** Values obtained from the proposed workflow for left coronary geometry is compared against values obtained from manual segmentation carried out using Vascular Modelling toolkit (VMTK)

	Length		
	RCA		
	Automatic	Manual	RE (×100)
Patient 6	6.11	9.97	0.39
Patient 14	11.44	10.30	0.11
Patient 23	11.28	14.09	0.20
Patient 24	11.09	13.99	0.21

**TABLE B7:** Initial vessel radius obtained from the proposed workflow for right coronary geometry is compared against values obtained from manual segmentation carried out using Vascular Modelling toolkit (VMTK)

	$R_{0}$		
	RCA		
	Automatic	Manual	RE (× 100)
Patient 6	0.92	1.72	0.47
Patient 14	1.63	1.89	0.14
Patient 23	1.75	1.26	0.39
Patient 24	1.78	1.39	0.28

**TABLE B8:** Final vessel radius obtained from the proposed workflow for right coronary geometry is compared against values obtained from manual segmentation carried out using Vascular Modelling toolkit (VMTK)

	*R*_f_		
	RCA		
	Automatic	Manual	RE (×100)
Patient 6	0.54	0.96	0.44
Patient 14	0.43	0.86	0.50
Patient 23	1.57	1.18	0.33
Patient 24	1.31	0.69	0.90

**TABLE B9:** Minimum vessel radius at stenosis location obtained from the proposed workflow for right coronary geometry is compared against values obtained from manual segmentation carried out using Vascular Modelling toolkit (VMTK)

	*R*_s_		
	RCA		
	Automatic	Manual	RE (×100)
Patient 6	0.41	0.53	0.23
Patient 14	0.47	0.51	0.08
Patient 23	1.06	0.92	0.15
Patient 24	1.45	0.84	0.73
